# The Transcaval AngioVac Aspiration of a Left Subclavian Artery Intimal Sarcoma Masquerading as Arterial Thrombosis: A Case Report

**DOI:** 10.3390/reports9030315

**Published:** 2026-09-18

**Authors:** Andrija Matetic, Frane Runjić, Nikola Crnčević, Goran Potočki, Antonija Sviličić, Ivona Mustapić, Darija Baković Kramarić

**Affiliations:** 1 Department of Cardiology, University Hospital of Split, 21000 Split, Croatiamateljanka@gmail.com (I.M.);; 2 Department of Cardiac Surgery, University Hospital of Split, 21000 Split, Croatia

**Keywords:** AngioVac, transcaval access, aspiration thrombectomy, intimal sarcoma, subclavian artery, case report

## Abstract

**Background and Clinical Significance:** Intimal sarcoma is a rare, aggressive malignancy of the large arteries that grows within the vessel lumen and frequently mimics thromboembolic disease, leading to diagnostic delay. Flow restoration and tissue biopsy are both difficult if the tumour occupies a technically demanding arterial segment, which is further aggravated if conventional large-bore arterial access is precluded by peripheral arterial disease. **Case Presentation:** A 66-year-old woman with multiple cardiovascular risk factors presented with subacute left upper-limb ischaemia and painless digital cyanosis (embolic phenomena) that had progressed despite therapeutic anticoagulation. Computed tomography angiography demonstrated a sub-occlusive filling defect extending from the origin of the left subclavian artery towards the aortic arch, initially interpreted as thrombus. There were no clear signs of malignant disease, despite differential screenings. Multiple on- and off-site surgical consultations were done, but the patient was repeatedly rejected for operation due to disease characteristics and high surgical risk. Severe iliofemoral peripheral arterial disease precluded usual transfemoral arterial delivery of a large-bore system, so the subclavian mass was aspirated using a 22/26-French sheath and extracorporeal aspiration system (AngioVac System (AngioDynamics)) delivered through percutaneous transcaval (caval–aortic) access, under cerebral embolic protection. Repeated aspiration runs with mechanical snare augmentation achieved substantial debulking, angiographic normalisation of flow and abolition of the invasive pressure gradient, without injury to the target artery. The caval–aortic tract was eventually closed with an occluder device. Histopathology of the aspirate revealed a malignant spindle-cell mesenchymal neoplasm consistent with intimal sarcoma. Staging subsequently demonstrated metastatic disease, and the patient proceeded to covered-stent maintenance of subclavian patency and first-line systemic chemotherapy. The patient is undergoing chemotherapy at 7-month follow-up, but palliation is being considered due to disease progression. **Conclusions:** Transcaval delivery of a large-bore aspiration system (AngioVac) is a feasible strategy to achieve both flow restoration and diagnostic tissue sampling when conventional arterial access is unavailable. Intimal sarcoma should be considered when arterial “thrombus” fails to respond to anticoagulation, particularly alongside constitutional symptoms.

## 1. Introduction and Clinical Significance

Intimal sarcoma is an exceptionally rare malignant mesenchymal tumour that arises from the intimal layer of large blood vessels of the systemic and pulmonary circulation, as well as the heart [1]. The incidence of intimal sarcoma is 0.001–0.03% [2]. It is characterised by intraluminal growth, an aggressive clinical course and a poor prognosis that is determined chiefly by the feasibility of complete surgical resection [1,3]. As it grows within the vessel lumen, it commonly produces obstruction and distal embolisation and, on cross-sectional imaging, appears as an intraluminal filling defect that is readily mistaken for bland thrombus [3,4,5]. This mimicry is a recurrent theme across reported cases in the aorta, pulmonary artery and cardiac chambers, and is a major cause of diagnostic delay [3,4,5].

Catheter-directed vacuum-assisted aspiration using an extracorporeal circuit (AngioVac System [AngioDynamics, 14 Plaza Dr, Latham, NY, USA]) has been applied to a widening range of intravascular material, including venous and right-sided cardiac tumours, endocarditis and thrombi [6]. More recently, there are emerging case reports utilising AngioVac in left-sided cardiac, arterial and aortic targets. The standard AngioVac System aspiration cannula comes in a 22-French shaft size with a tip that expands up to 42–48 French. It requires a 26-French sheath for successful insertion. Beyond its therapeutic role in restoring flow, aspiration can provide tissue that enables pathohistological or microbiologic diagnosis. Delivery of such extra-large-bore systems, however, ordinarily requires substantial arterial or venous access, which may be unavailable in patients with severe peripheral arterial disease [7].

Transcaval (caval–aortic) access was developed to enable fully percutaneous delivery of large-calibre devices into the abdominal aorta from the femoral vein in patients who are ineligible for transfemoral arterial access [7]. The technique is well established for transcatheter aortic valve replacement and other large-bore aortic interventions [7].

To the best of our knowledge, we describe the first ever use of a transcaval route to deliver a large-bore aspiration system for the treatment and diagnosis of an arterial (left subclavian) intimal sarcoma in a patient in whom large-bore transfemoral arterial access was precluded by severe iliofemoral disease.

## 2. Case Presentation

### 2.1. Presentation and History

A 66-year-old woman presented with a several-week history of pain in the left hand and the radial aspect of the left forearm, initially exertional and subsequently present at rest. She had a long history of heavy cigarette smoking (~94 pack years), arterial hypertension, type 2 diabetes mellitus and dyslipidaemia, and reported unintentional weight loss of 7–8 kg over the preceding months. Approximately 7 weeks before admission she had been started on therapeutic low-molecular-weight heparin by a vascular surgeon for a presumed thrombotic occlusion of the left subclavian artery. Despite anticoagulation she developed painless cyanosis of the left fourth and fifth finger, consistent with ongoing ischaemia and distal embolisation. She was admitted to a regional hospital and transferred to our tertiary cardiovascular centre for further evaluation and treatment.

On examination she was haemodynamically stable and afebrile. A striking finding was marked interarm blood-pressure asymmetry (left arm 69/55 mmHg versus right arm 122/82 mmHg) with a weakly palpable left radial pulse, in line with a proximal left subclavian obstruction. There was livedo reticularis of the upper limbs and dusky discoloration of the left fingers, most marked at the fourth and fifth fingertips, with features of established and subacute ischaemic injury.

### 2.2. Investigations

**Figure 1:**
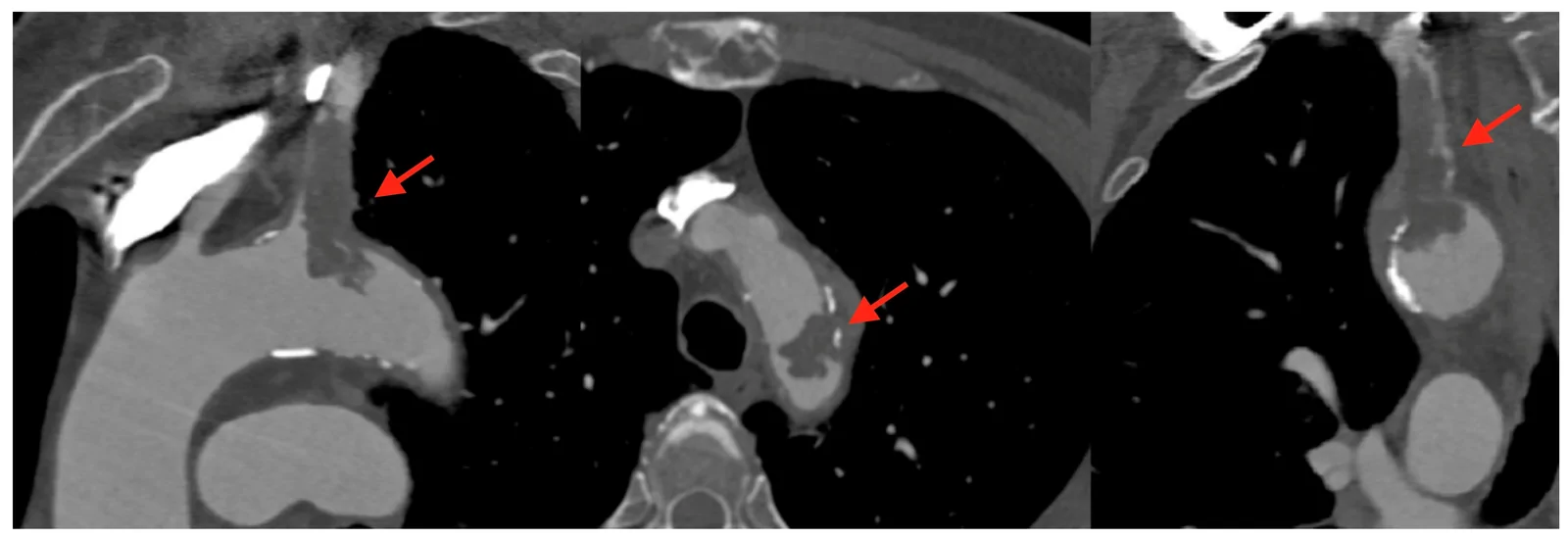
Computed tomography angiography at presentation. Representative multiplanar reconstruction showing the sub-occlusive intraluminal filling defect extending from the left subclavian artery (arrow) with extension towards the aortic arch, initially interpreted as thrombus.

Computed tomography angiography of the aorta demonstrated a filling defect extending from the origin of the left subclavian artery over a length of ~3.1 cm, with a near-complete luminal occlusion and only a thin peripheral rim of contrast opacifying the more distal subclavian artery. Importantly, the abnormal intraluminal material was noted to extend towards the aortic arch (Figure 1). The index MSCT scanning report described only a few non-specific pulmonary nodules, without any bone anomalies. Small pulmonary nodules were non-specific and not available for tissue biopsy.

Transthoracic echocardiography showed a mildly reduced left-ventricular ejection fraction (~44%) with reduced global longitudinal strain and otherwise competent valves. An extensive search for an underlying cause of arterial thrombosis and embolisation was largely unrevealing. A panel of serum tumour markers (including CEA, CA 19-9, CA 15-3, CA 125, NSE and CYFRA 21-1) was within normal limits; antiphospholipid antibodies were negative; and antinuclear antibodies were positive at a titre of 1:160 with an otherwise unremarkable extractable-nuclear-antigen profile. Other thrombophilia, vasculitis and infectious screens did not identify a causative disorder, although serology indicated past hepatitis B infection. The combination of an embolising “thrombus” that had progressed on therapeutic anticoagulation, together with unexplained constitutional weight loss, raised concern that the subclavian lesion might not be a bland thrombus.

### 2.3. Endovascular Procedure: Transcaval AngioVac Aspiration

**Figure 2:**
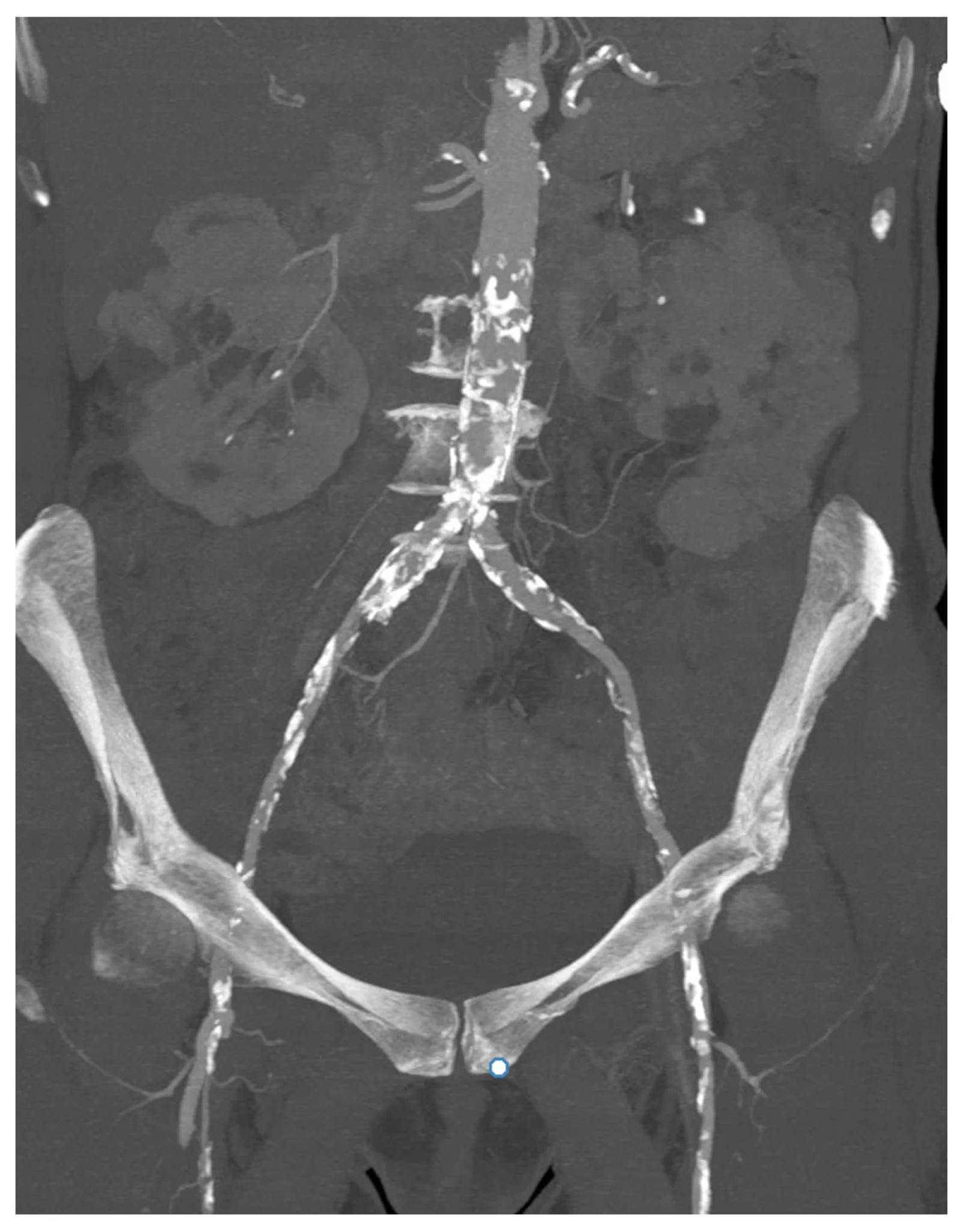
Computed tomography angiography of iliofemoral arteries.

After multidisciplinary Heart Team discussion, the patient was deemed at prohibitive risk for complex surgical operation. Therefore, transcatheter aspiration of the sub-occlusive left subclavian mass was planned. The patient’s severe iliofemoral peripheral arterial disease precluded safe delivery of a large-bore 26-French sheath and 22-French aspiration system through a transfemoral arterial route [6] (Figure 2). Therefore, a transcaval (caval–aortic) approach was selected to deliver the system into the aorta from the venous side, as it was the only possible access for such large equipment (Supplementary Video S1).

**Figure 3:**
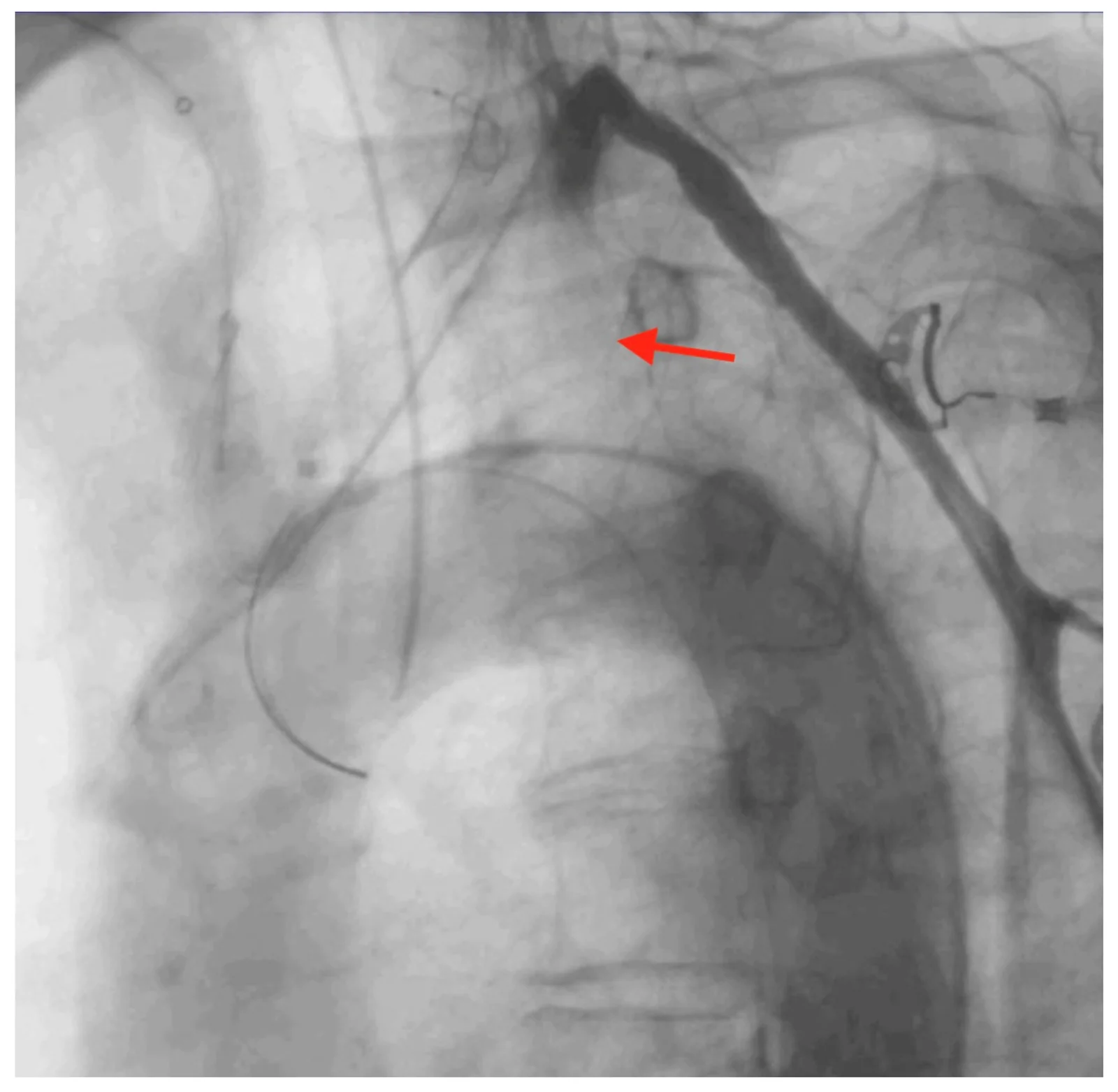
Initial digital subtraction angiography during procedure. Figure showing the sub-occlusive intraluminal filling defect extending from the left subclavian artery (arrow). Cerebral embolic protection in situ.

Following a detailed multimodality planning, reconstruction and simulation, the endovascular procedure was undertaken under general anaesthesia with perfusion support. Cerebral embolic protection was first established through right transradial access, with a dual-filter device deployed to protect the brachiocephalic and left common carotid arteries given the proximity of the lesion to the arch and the planned manipulation near the supra-aortic vessels. A pigtail catheter was placed through left transradial access for haemodynamic monitoring and angiography (Figure 3).

**Figure 4:**
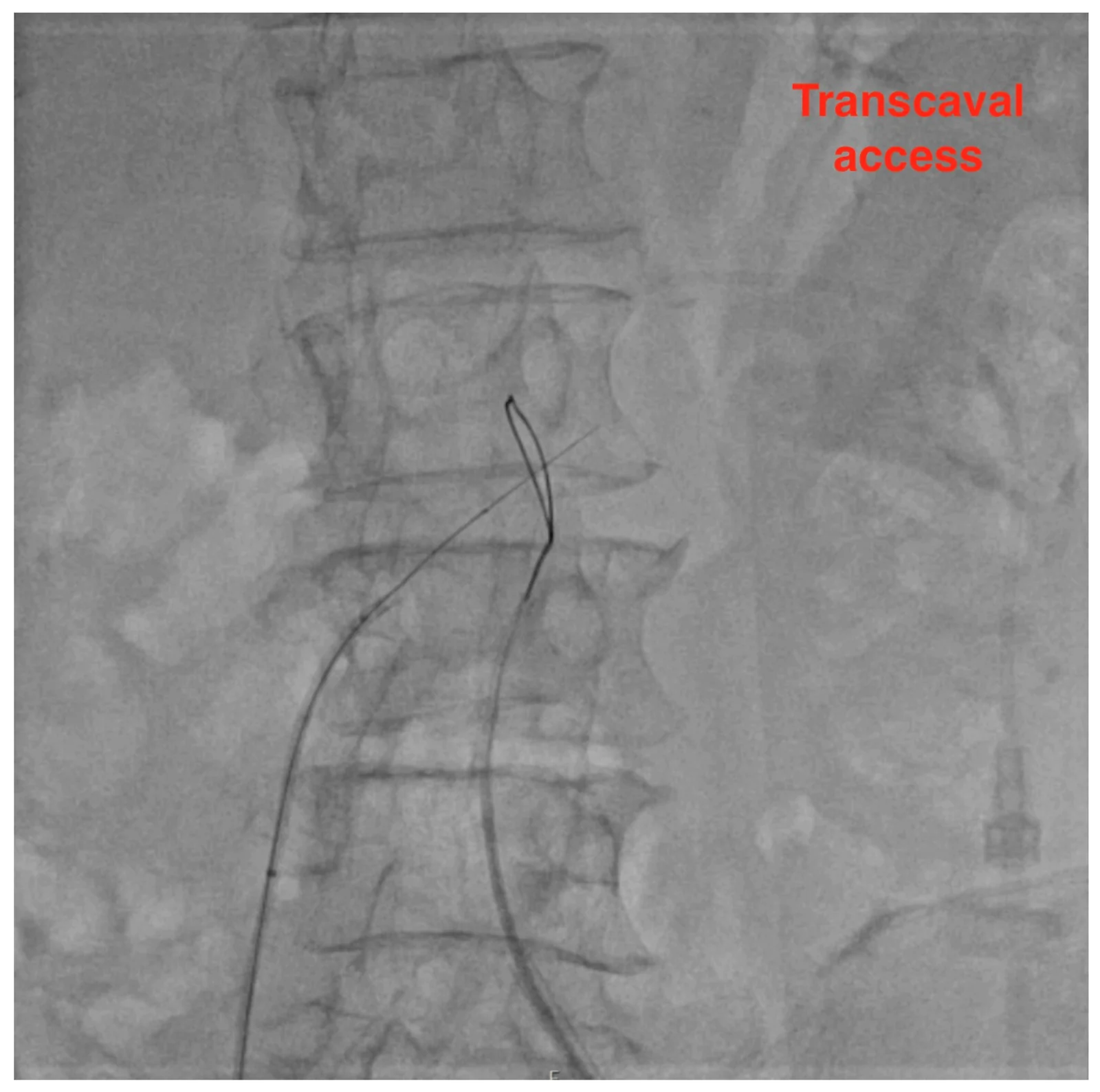
Fluoroscopic view of the transcaval crossing.

Bilateral transfemoral arterial access was obtained. The right femoral artery was used for 17-French an arterial return cannula, while the left femoral artery was used as a secondary access for transcaval entry. The right femoral vein was the main access for 26-French transcaval entry and AngioVac delivery (Figure 4; Supplementary Videos S2 and S3).

**Figure 5:**
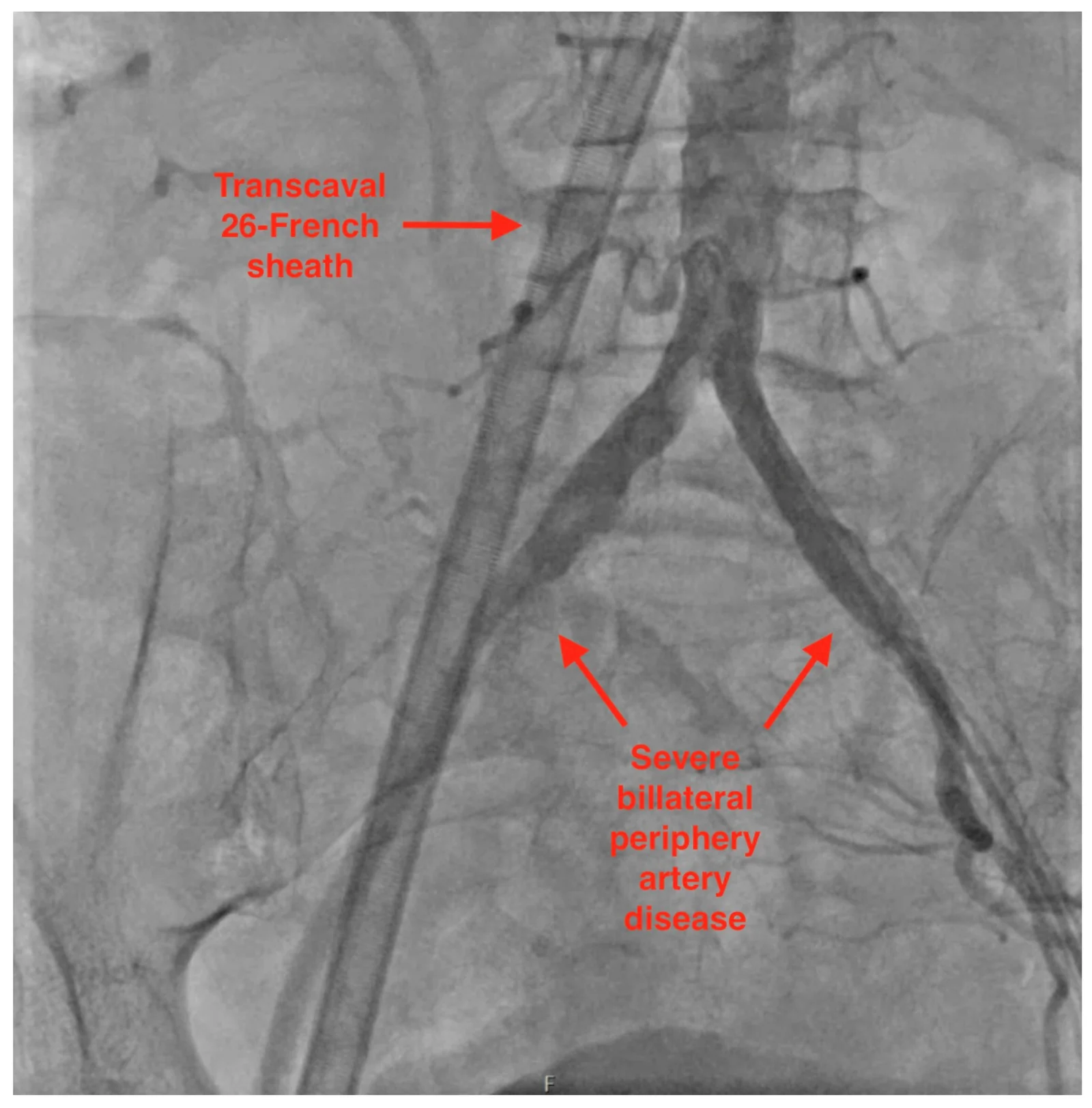
Fluoroscopic view showing the size discrepancy of small diseased iliofemoral arteries and large 26-French transcaval sheath.

A large-bore introducer sheath (26-French) was advanced across the tract, through which a 22-French aspiration cannula (AngioVac) was positioned at the ostium of the left subclavian artery (Figure 5).

**Figure 6:**
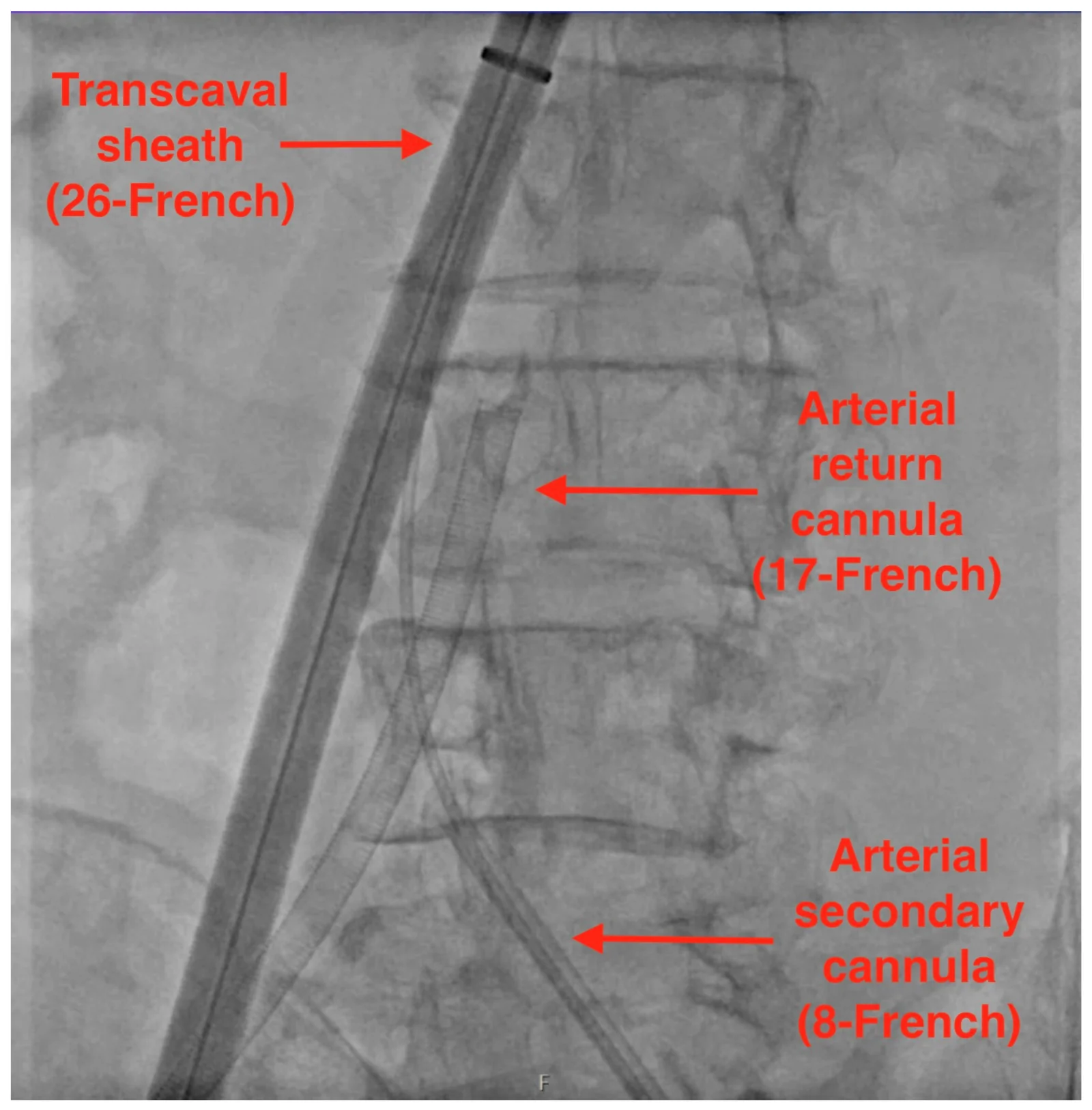
Fluoroscopic view showing the peripheral cannulas: transcaval 26-French sheath; arterial 17-French circuit return cannula; arterial 8-French secondary cannula.

An arterial return cannula (17-French) was placed in the right common femoral artery, and an arterio-arterial extracorporeal circuit was established (Figure 6).

**Figure 7:**
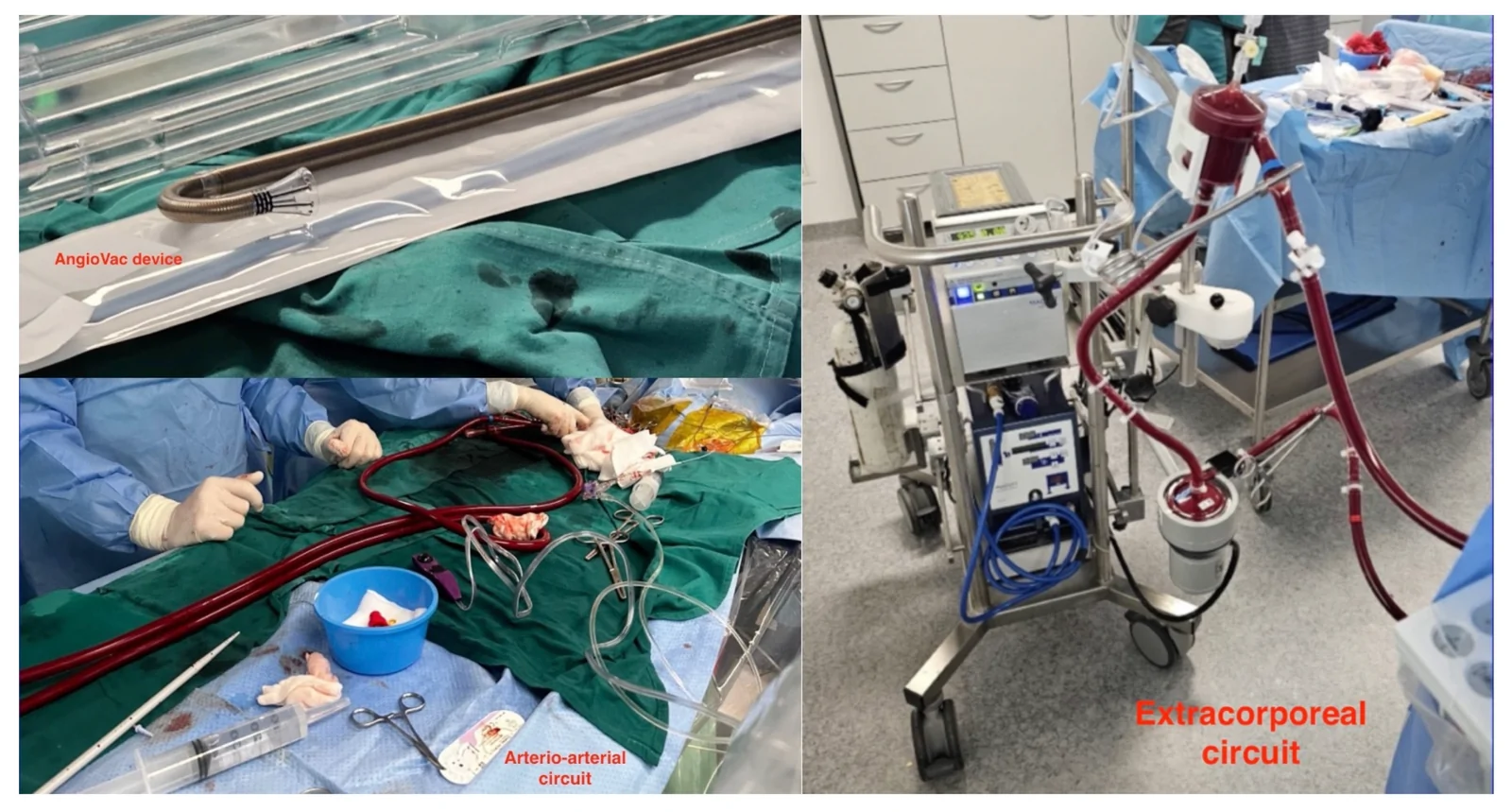
Procedural set-up: aspiration cannula with funnel-like end (AngioVac 22F); table set-up showing the extracorporeal arterio-arterial circuit cannulas; extracorporeal device allowing for arterio-arterial circulation.

An arterio-arterial circuit with extracorporeal circulation was established using the extracorporeal centrifugal pump (RotaFlow; Maquet Cardiopulmonary AG, Rastatt, Germany) (Figure 7).

**Figure 8:**
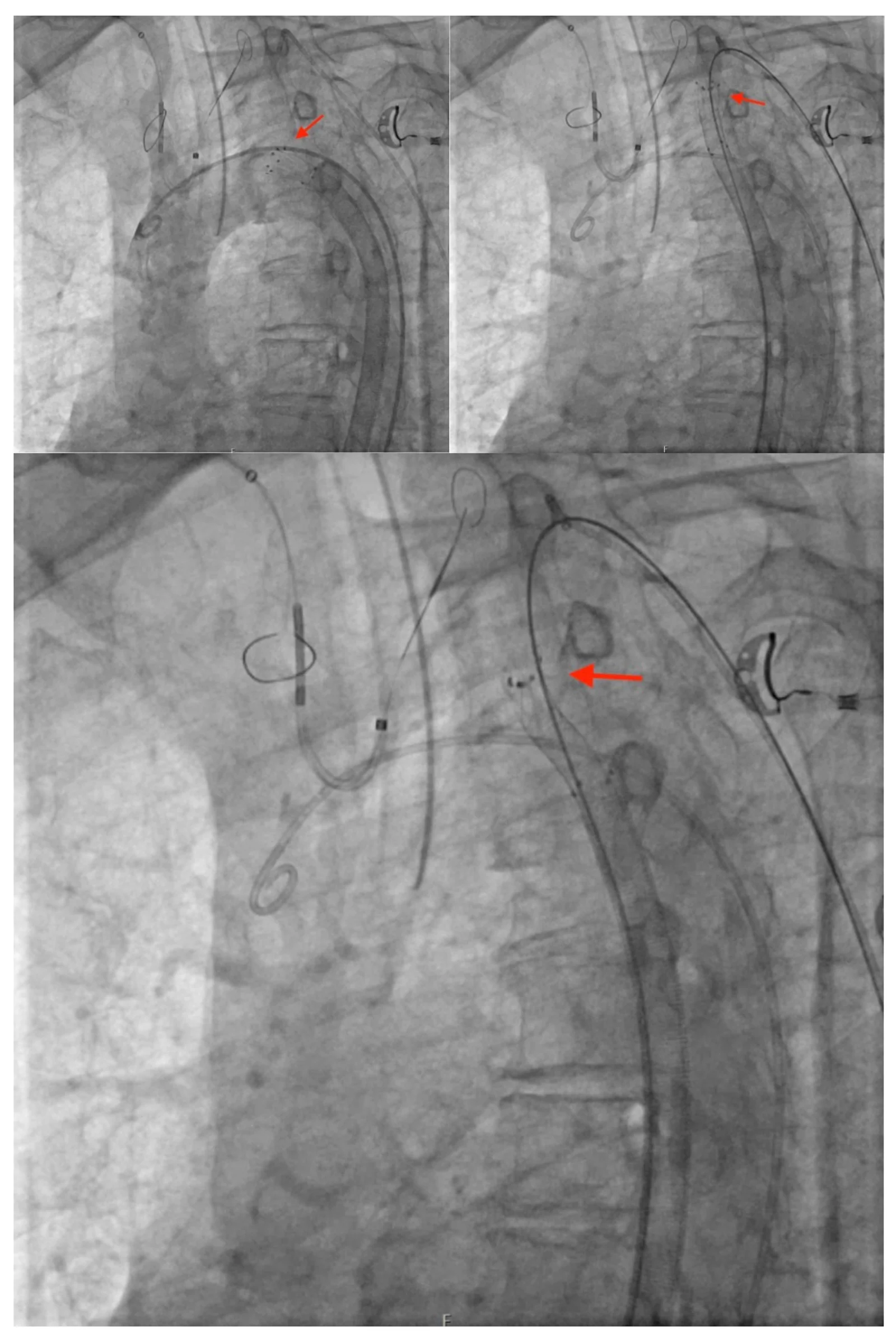
Aspiration with AngioVac 22F: deep inside left subclavian artery.

Multiple controlled aspiration runs were performed (up to 4500 rpm), with simultaneous mechanical augmentation using a snare to mobilise the mass (Figure 8; Supplementary Videos S4 and S5).

Aspiration achieved removal of a substantial portion of the intraluminal mass, with angiographic normalisation of flow in the left subclavian artery and abolition of the invasive trans-lesional pressure gradient, and without injury to the target artery. The cerebral protection filters were noted to contain captured particulate material on retrieval, underscoring the embolic potential of the lesion. The extracorporeal circuit and the cerebral protection device were removed.

**Figure 9:**
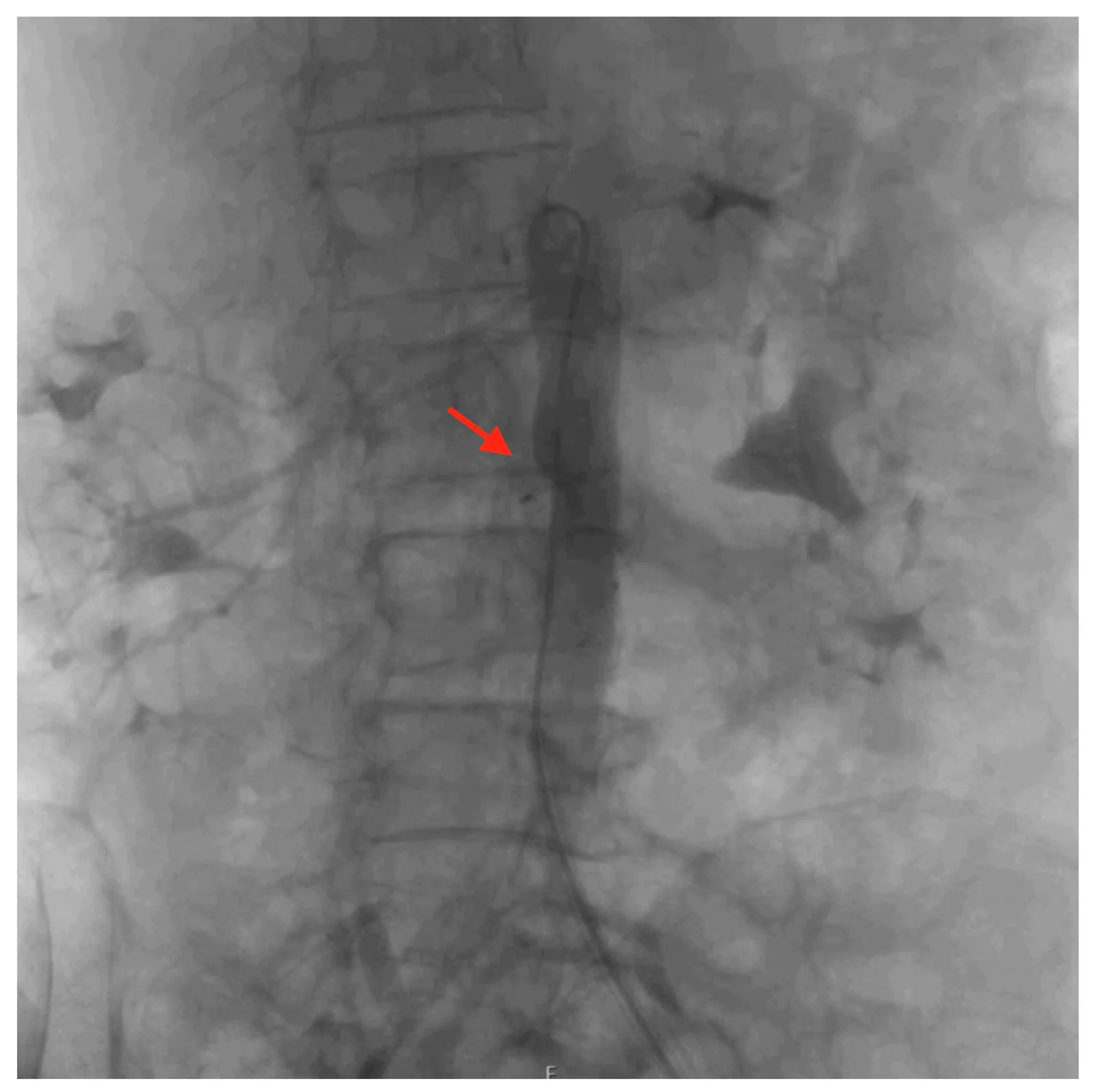
Successful closure of transcaval tract (type 0 closure).

The caval–aortic tract was closed with a nitinol duct occluder (Amplatzer Duct Occluder 1 (12/10); 5050 Nathan Lane N, Plymouth, Minenesota, USA) with brief dry haemostasis (adjunctive balloon inflation), achieving complete occlusion of the tract (type 0 closure) (Figure 9; Supplementary Videos S6, S7, S9 and S10). Arterial and venous access sites were closed with vascular closure devices, and haemostasis was confirmed by ultrasound and angiography.

**Figure 10:**
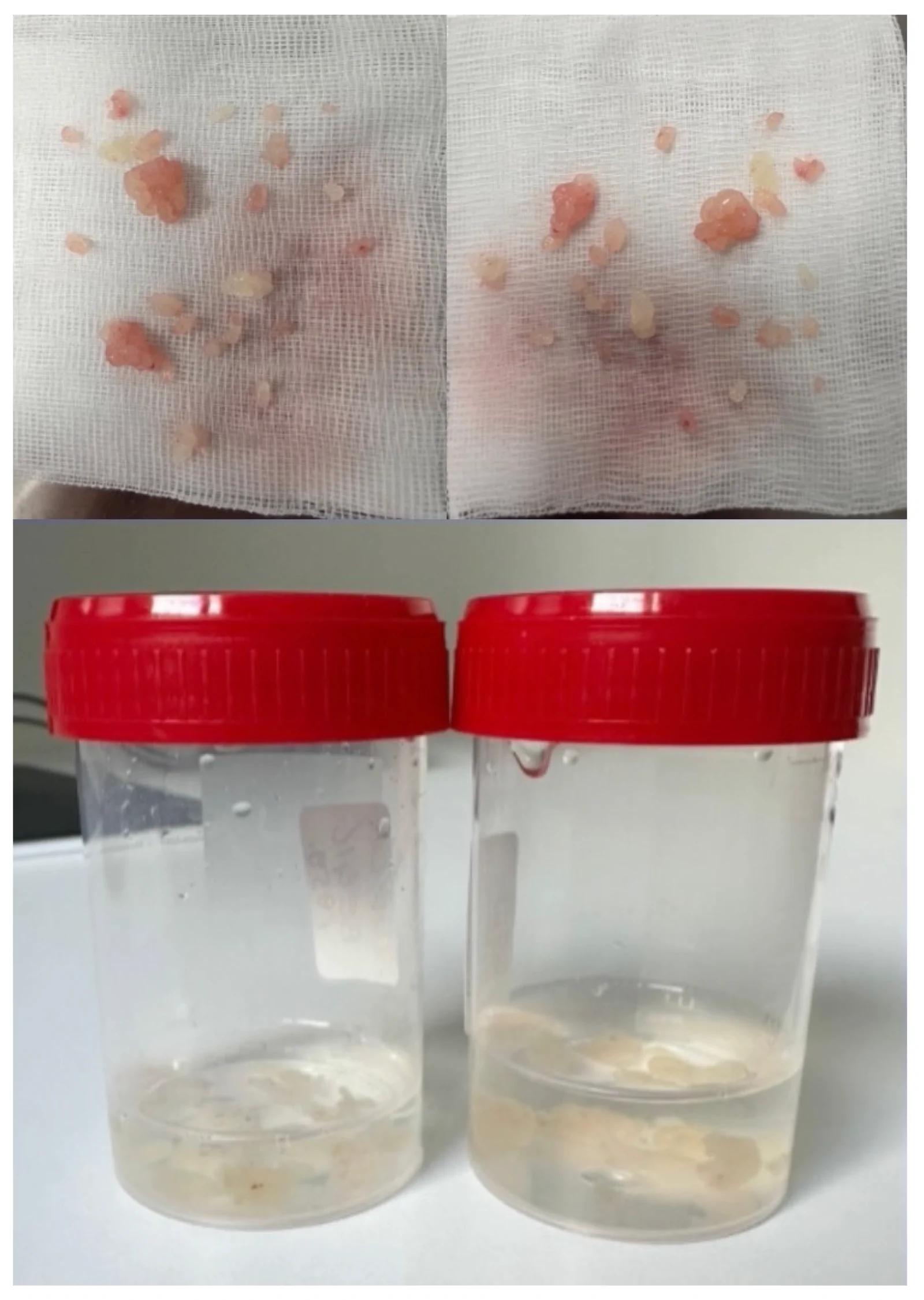
Multiple tumour samples.

The evacuated material was sent for histopathological analysis (Figure 10). The patient was transferred to the cardiovascular intensive care unit for routine observation, and control cross-sectional imaging was performed the following day.

### 2.4. Histopathology

The aspirated material consisted of multiple small, whitish tissue fragments that were embedded in their entirety in two paraffin blocks. Histology showed hypercellular tumour tissue composed of monotonous atypical spindle cells set in a myxoid stroma that was Alcian-blue-positive; tumour cellularity was denser towards the surface, and part of the surface showed an immunohistochemically demonstrable endothelial lining (factor VIII-positive). Immunohistochemically, the tumour cells were diffusely positive for calponin and weakly positive for actin, with focal positivity for CD31 and oestrogen receptor, and were negative for CD34, S100, pan-cytokeratin, desmin, MyoD1, h-caldesmon, calretinin, CD117 and p16; p53 expression was heterogeneous and the Ki-67 proliferation index was approximately 25%. EBER in situ hybridisation was negative, and FISH analysis showed no amplification of the *MDM2* gene. The findings were those of a malignant mesenchymal neoplasm; in view of the location, morphology and immunophenotype, intimal sarcoma was considered the leading diagnosis, and molecular analysis by next-generation sequencing was recommended.

### 2.5. Outcome and Follow-Up

**Figure 11:**
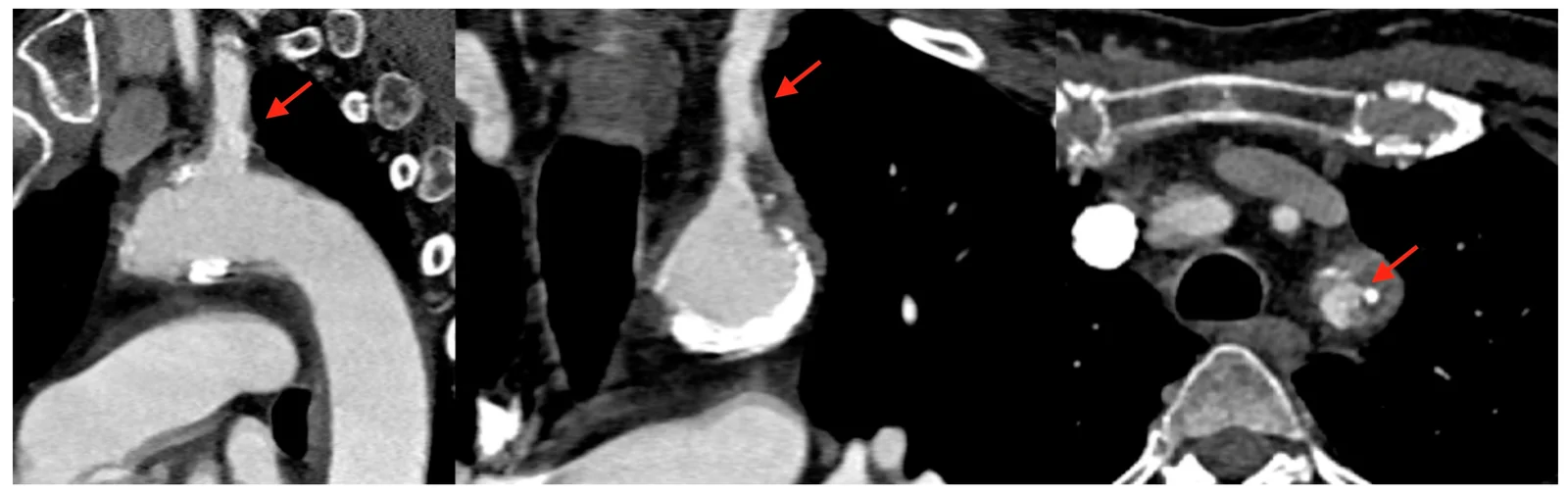
Computed tomography angiography after the procedure. Representative multiplanar reconstruction showing substantial resolution of stenosis within the left subclavian artery (arrow).

Control imaging after the procedure confirmed a good technical result, with substantial reduction in the intraluminal mass and satisfactory antegrade flow in the left subclavian artery (Figure 11), accompanied by clinical improvement of the left hand and a reduction in the interarm blood-pressure difference. The patient was discharged on antithrombotic and cardiovascular secondary-prevention therapy.

Definitive histopathology, available approximately 3 weeks after the procedure, confirmed intimal sarcoma. Staging undertaken about 6 weeks after the index aspiration demonstrated metastatic disease, with osteolytic skeletal deposits, in line with the rapidly progressive intimal sarcoma disease. Furthermore, there was a persistent tumoral thickening (approximately 3.5 × 2.5 cm) causing recurrent subtotal ostial occlusion of the left subclavian artery. Because of the embolic digital ischaemia, partial amputation of the left fifth finger was eventually required, with ongoing ischaemic changes in the third and fourth fingertips.

The patient was subsequently managed by the multidisciplinary team. To maintain arterial patency across the tumour-bearing ostial segment, two overlapping covered stents (stent grafts) were deployed across the left subclavian origin, with angiographic restoration of flow and no dissection or distal embolisation. A totally implantable venous access port was placed, and first-line systemic chemotherapy for metastatic sarcoma (a doxorubicin–ifosfamide regimen) was commenced; the expected chemotherapy-induced myelosuppression (neutropenia, anaemia and thrombocytopenia) at nadir was managed supportively. The patient was discharged in an improved general condition (ECOG performance status 1–2), with a plan for continued chemotherapy, restaging imaging after three cycles, and further molecular characterisation (a liquid biopsy was planned after the non-diagnostic tissue biopsy).

## 3. Discussion

This case illustrates several important points: first, the propensity of intimal sarcoma to masquerade as arterial thrombus; second, the value of catheter-directed aspiration in simultaneously restoring flow and providing diagnostic tissue; and third, the utility of transcaval access when severe peripheral arterial disease precludes conventional large-bore arterial delivery. Contrary to previous transcaval AngioVac reports which targeted aortic thrombi, this case is the first to address an arterial intimal sarcoma [8,9]. The patient is undergoing chemotherapy at 7-month follow-up, but palliation is being considered due to signs of disease progression.

As noted, this intraluminal-growth pattern mimics bland thrombus, which delayed recognition in this case scenario [1,4,5]. The most useful clinical clue in the present case was the failure of a presumed subclavian “thrombus” to respond to therapeutic anticoagulation, with continued distal embolisation, occurring together with unexplained constitutional weight loss. A lesion that progresses or continues to embolise despite adequate anticoagulation should prompt consideration of an intraluminal neoplasm, and multimodality imaging (including the metabolic activity of the lesion on PET where available, and gadolinium enhancement on MRI) can help distinguish tumour from thrombus. However, these investigations are non-specific and have diagnostic limitations [10], often warranting further procedures such as surgical removal with ex tempore analysis. In this specific case scenario, an inclusion of additional non-invasive imaging modalities could have provide earlier data that it was an underlying malignancy, but treatment of aortic protruding mass and ischaemic arm (even if palliation) would require a similar therapeutic approach.

From a diagnostic standpoint, the percutaneous aspirate in this case was decisive: material presumed to represent thrombotic occlusion proved instead to be a malignant spindle-cell neoplasm. A comparable “thrombus-versus-tumour” clarification through aspiration has been reported for intracardiac masses, where AngioVac achieved both a reduction in mass burden and the correct diagnosis [6]. The immunophenotype in our case—diffuse calponin with focal CD31 and a focal factor VIII-positive surface, and negativity for h-caldesmon, S100, pan-cytokeratin and desmin—supported a poorly differentiated intimal sarcoma. The diagnostic yield of aspiration proved particularly valuable in this case, but also provided initial therapeutic relief.

The transcaval access strategy was the pivotal technical decision and only possible option [7]. Repurposing the transcaval route to deliver an aspiration system to an arterial target extends its application beyond device implantation to catheter-directed thrombectomy/tumour aspiration [8,9]. Furthermore, the utilisation of cerebral embolic protection proved to be reasonable due to captured particles.

Several caveats apply. Aspiration is a debulking and diagnostic manoeuvre rather than an oncological resection. In this patient, later staging after diagnosis revealed metastatic (skeletal and pulmonary) disease, and further management accordingly was palliative, comprising systemic chemotherapy together with covered-stent local control of the tumour-bearing subclavian segment. This course reflects the poor prognosis of intimal sarcoma, which is determined primarily by the feasibility of complete surgical excision, so that multimodality treatment is generally required and the durable oncological benefit of an endovascular debulking approach is unknown. Transcaval access carries its own risks, principally bleeding and access-site vascular complications, and demands appropriate planning, imaging and operator experience. Therefore, it should be performed only in experienced centres with high-volume expertise.

## 4. Conclusions

This is the first case report of a successful transcaval large-bore aspiration (AngioVac) of a sub-occlusive tumorous mass (intimal sarcoma) of the left subclavian artery, achieving diagnostic and therapeutic benefits, and guiding further management. When conventional large-bore arterial access is precluded by severe peripheral arterial disease, transcaval delivery of a large-bore aspiration system is a feasible option. Intimal sarcoma should be considered whenever an arterial “thrombus” fails to respond to adequate anticoagulation, continues to embolize, or is accompanied by constitutional symptoms such as unexplained weight loss.

## Data Availability

The data supporting the findings of this case report are contained within the article. Further enquiries can be directed to the corresponding author.

## Supplementary Materials

The following supporting information can be downloaded at: https://www.mdpi.com/article/10.3390/reports9030315/s1, Videos S1–S7, S9 and S10.
